# An adult case of invasive pneumococcal disease due to serotype 12F-specific polysaccharide antibody failure following a 23-valent polysaccharide vaccination

**DOI:** 10.1080/22221751.2020.1830716

**Published:** 2020-10-14

**Authors:** Yasuhiro Tanaka, Kazuko Yamamoto, Yuichi Fukuda, Asuka Umemura, Masataka Yoshida, Shuhei Ideguchi, Nobuyuki Ashizawa, Tatsuro Hirayama, Masato Tashiro, Takahiro Takazono, Yoshifumi Imamura, Taiga Miyazaki, Koichi Izumikawa, Katsunori Yanagihara, Bin Chang, Hiroshi Mukae

**Affiliations:** a Department of Respiratory Medicine, Nagasaki University Hospital, Nagasaki City, Japan; b Department of Respiratory Medicine, Sasebo City General Hospital, Sasebo City, Japan; c Infection Control and Education Center, Nagasaki University Hospital, Nagasaki City, Japan; d Department of Laboratory Medicine, Nagasaki University Hospital, Nagasaki City, Japan; e Department of Bacteriology I, National Institute of Infectious Diseases, Shinjuku City, Japan

**Keywords:** Pneumococcal vaccine, *Streptococcus pneumoniae* infection, *Streptococcus pneumoniae* serotype 12 F, invasive pneumococcal disease, opsonophagocytosis assay

## Abstract

A 68-year-old Japanese man was admitted to our hospital for an acute febrile illness with shivering and impaired consciousness. He was a previous smoker and had a history of chronic obstructive pulmonary disease, for which he inhaled steroid with a long-acting bronchodilator. He had received a 23-valent pneumococcal polysaccharide vaccination 2 years previously. He was intubated and placed on a ventilator in intensive care unit because of acute respiratory failure and hypercapnia. Streptococcus pneumoniae was grown from his blood, sputum, and urine cultures, and he was diagnosed with invasive pneumococcal disease with acute renal failure. He was treated with intravenous beta-lactam and macrolide with continuous hemodiafiltration and was discharged 3 months later. The pneumococcus was identified as serotype 12F, and his serotype-specific IgG and opsonophagocytic index against serotype 12F indicating a lack of protection from IPD among PPV23 serotypes. This case highlights that some individuals may have a serotype-specific polysaccharide antibody failure that makes them susceptible to serotype 12F invasive pneumococcal disease. This case also illustrates the need for serotype-specific IgG and opsonophagocytic index titre cut-offs for each specific pneumococcal serotype in available vaccines to understand the vaccination protection for individual patients better.

We describe a case of invasive pneumococcal disease (IPD) due to serotype 12F-specific antibody functional failure determined by opsonophagocytosis assay in a man who received a 23-valent polysaccharide vaccination.

A 68-year-old Japanese man was admitted to our hospital for an acute febrile (37.9°C) illness with shivering and impaired consciousness. He was a previous smoker and had a four-year history of chronic obstructive pulmonary disease, for which he inhaled steroid with a long-acting bronchodilator. He had received a 23-valent pneumococcal polysaccharide vaccination (PPV23) 2 years previously. On admission, he had an acute respiratory failure (PaO_2_/FiO_2_: 52 mmHg) and hypercapnia (PaCO_2:_ 57 mmHg). His laboratory test results showed raised C-reactive protein of 255 mg/L (reference: <1.4 mg/L) and procalcitonin of 293 ng/L (reference: 4.9 ng/L) with mild leucocytosis (white blood cell count 8,740 cells/μL, reference range: 3,300–8,600 cells/μL), neutrocytosis (97.3%), and thrombocytopenia (platelet count: 95,000/μL). Serum immunoglobulin (Ig) levels were normal (IgA, 211 mg/dL [reference range: 93–393 mg/dL]; IgM, 65 mg/dL [reference range: 33–183 mg/dL]; and IgG, 1,256 mg/dL [reference range: 861–1,747 mg/dL]). Blood urea nitrogen level was 97.2 mg/dL and creatinine, 3.61 mg/dL, with microhaematuria, indicating acute renal failure. His disseminated intravascular coagulation score was 3, and his Sequential Organ Failure Assessment score was 10. He was admitted to the intensive care unit, where he was intubated and placed on a ventilator. Chest radiograph showed extensive consolidation in the left lung (Figure 1, Panel A), and abdominal chest computed tomography showed dense consolidation with lobar pneumonia in the left lung with mild left pleural effusion (Figure 1, Panel B). His spleen size was normal. Blood, sputum, and urine cultures showed *Streptococcus pneumoniae*. He was diagnosed with bacteraemic IPD with pneumonia and acute renal failure. Intravenous meropenem and azithromycin and continuous hemodiafiltration were initiated. On Day 7, susceptibility testing revealed that *S. pneumoniae* was susceptible to penicillin; therefore, the antibiotics were switched to sulbactam/ampicillin. He was extubated on Day 24; the antibiotics were stopped after 31 days. His consciousness level returned to normal, and he was discharged on Day 92 with home oxygen therapy. Later, the pneumococcus was identified as serotype 12F by the Quellung reaction and was determined as sequence type (ST) 6945 by multilocus sequence typing, both conducted at the National Institute of Infectious Diseases. To determine the protective immunity to the infecting serotype, we examined serotype-specific IgG levels and opsonophagocytic index (OI) using a multiplexed opsonophagocytic assay as previously described (see http://www.vaccine.uab.edu) [1]. Among PPV23 serotypes, his serotype-specific IgG and OI [2] against serotype 12F were 3.42 μg/mL and <4, respectively (Figure 1, Panel C), indicating a lack of protection from IPD [3–5]. However, OIs against the other serotypes tested were in the acceptable range (Figure 1, Panel C).

**Figure 1.**
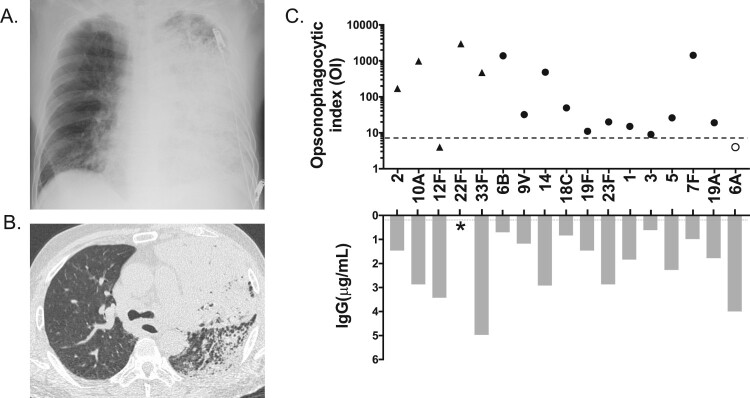
Panel A. Supine anteroposterior chest radiograph on admission. An extensive consolidation is seen in the left lung. Panel B. Axial chest computed tomography image on admission. Multiple and diffuse low attenuation areas in bilateral lungs represent emphysema. Lobar consolidation is seen in the left upper lobe. Bronchial wall thickness and partial consolidation are seen in the left lower lobe. Panel C. Opsonophagocytic index (OI) and IgG of the patient’s serum for independent pneumococcal serotypes. A dotted line represents a cut-off OI of 8 (3). A grey line represents a cut-off of IgG of 0.2 μg/mL (5). Filled circles represent both PSV23- and PCV13-covered serotypes. Filled triangles represent only PSV23-covered serotypes. An open circle represents only the PCV13-covered serotype. Serotype 12F is the only serotype that was below the threshold in PSV23-covered serotypes. *An IgG against serotype 22F is missing due to the WHO-approved ELISA standard procedure (4).

*S. pneumoniae* serotype 12F is rarely found among colonising isolates. Nevertheless, IPD incidence in children and adults, caused by serotype 12F, has noticeably increased internationally since the introduction of the 7- and 13-valent pneumococcal conjugate vaccines (PCVs) [6, 7]. ST 6945, the sequence type detected in our case, is the second major typing following ST 4846, in the surveillance of adult IPD caused by *S. pneumoniae* 12F in Japan [8]. The PPV23 vaccine covers the 23 most common capsule types that cause IPD. It was introduced in the routine immunisation schedule in Japan in October 2014 and is recommended for adults ≥ 65 years and for those aged 60–64 years with cardiac, renal, or respiratory dysfunction or immunodeficiencies [9]. A splenectomised woman developed recurrent IPD due to *S. pneumoniae* 12F after vaccination with PPV23 owing to low avidity of serotype-specific antibody [5, 10], and adults with asplenia/hyposplenia or who underwent splenectomy have increased fatality rates due to *S. pneumoniae* 12F IPD compared with other serogroups [8]. Both suggest that the functional activity of serotype 12F-specific antibodies is a key factor for preventing IPD caused by *S. pneumoniae* 12F. Though the spleen size of this patient was normal, the moderate antibody production against serotype 12F was higher than 0.2 µg/mL but lower than 5 µg/mL (5) in this patient, which might have been suppressed by his use of inhaled steroids because approximately 20% of patients with chronic pulmonary diseases are non-responders to specific serotypes for PPV23 [11]. Although no standard guidelines are available regarding serotype-specific OI titre cut-offs required for protecting adults against IPD after immunisation, an OI threshold of 8 serves as a guide for possible protection [3–5]. The PPV23 vaccine effectiveness to serotype 12F among older adults is generally high [12]; however, this case highlights that some individuals may have a serotype-specific polysaccharide antibody failure that makes them susceptible to serotype 12F IPD. This case also illustrates the need for serotype-specific IgG and OI titre cut-offs for each specific pneumococcal serotype in available vaccines to understand the vaccination protection for individual patients better.
